# How and when leader narcissism links to employees’ objective career success: The roles of ingratiation and careerist orientation

**DOI:** 10.3389/fpsyg.2022.1058018

**Published:** 2023-01-09

**Authors:** Zhihui Ding, Mingwei Liu, Lei Quan, Huaqiang Wang, Pengcheng Zhang, Wenxing Liu

**Affiliations:** ^1^College of Law and Public Administration, China Three Gorges University, Yichang, China; ^2^School of Management, Huazhong University of Science and Technology, Wuhan, China; ^3^School of Economics and Management, Yangtze University, Jingzhou, China; ^4^School of Business Administration, Zhongnan University of Economics and Law, Wuhan, China

**Keywords:** leader narcissism, ingratiation, careerist orientation, objective career success, dramaturgical theory

## Abstract

Previous researches have emphasized the value of leader narcissism on employees’ career success, whereas we still know little about how and when this relationship will materialize. By integrating dramaturgical theory and leader narcissism literatures, we propose a theoretical model to explain the mechanism and boundary of leader narcissism in promoting employees’ objective career success (e.g., salary increases and promotions). To test our hypotheses, we carried out a multi-wave research design and collected data from 299 employees in Chinese manufacturing firms. The results of multiple regression analysis showed that leader narcissism motivates employees’ ingratiation, which in turn facilitates employees’ objective career success, especially when those employees are high in careerist orientation. Theoretical and practical implications of the findings are discussed.

## Introduction

As a typical dark personality trait, narcissism implies arrogance, hostility, exaggeration and self-absorption (Rosenthal and Pittinsky, 2006). Due to the importance of leadership, leader narcissism has been widely discussed in previous studies (Gauglitz et al., 2022). Leader narcissism is perceived as leaders with narcissistic personality traits, and could be leading in an autocratic, exploitative and self-serving manner, thereby displaying unethical, despotic leadership (Grijalva et al., 2015), which is completely opposite of the positive leadership style such as charismatic leadership (Scardigno et al., 2021). The detrimental effect of leader narcissism on individuals have been extensively proved (Braun et al., 2018), such as inhibiting employees’ OCB (Wang et al., 2021), reducing employees’ job engagement (Fehn and Schütz, 2021) and job performance (Liu et al., 2022). They also tend to attribute the achievements of employees to themselves, even believing that it is appropriate for them (Aquino et al., 2006). Narcissistic leaders, however, are not always self-centered. They need to be recognized by their colleagues, especially their employees, to satisfy their own self-image and self-view (Morf and Rhodewalt, 2001). More recently, Volmer et al. (2016) proved that leader narcissism has a significant positive impact on employees’ career success which can greatly meet leaders’ internal needs for appreciation and exaggeration. Nevertheless, how and when will this relationship occur?

To address this question, we draw upon dramaturgical theory to investigate how and when leader narcissism positively predicts employees’ career success (Goffman, 1959). According to dramaturgical theory, we liken the workplace to a theater where individuals perform according to specific theatrical scenes and character norms to achieve personal goals. For employees, they can send a signal to their leaders that they are loyal to the role when they behave in accordance with the requirements of the role. Accordingly, leaders will also send signal which is important for employees to senders when they received signals from employees, which is an important commitment to each other for future dramas to continue. What is most important is that leaders can provide career development opportunities and contribute to the professional success for employees.

We propose that employees’ ingratiation tactics is an interactive and actor’s performance behavior that accesses resources, and the performance result is individuals’ income, namely, objective career success which is referred to material benefits such as salary increases and promotions (Goffman, 1959). Therefore, we identify employees’ ingratiating behavior as a critical intermediate process through which leader narcissism affects employees’ career success. Furthermore, dramaturgical theory posits that the intensity of dramatic behavior varies with performers’ personal goals, implying that the performers’ values will affect their performance on the stage (Wang and Chen, 2022), such as careerist orientation, which is referred to the tendency of employees to pursue career development in a non-performance-oriented way (Feldman and Weitz, 1991). Employees with careerist orientation are particularly interested in career success (e.g., promotions), and they attach great importance to the application of impression management strategies (Wu et al., 2013). It is important to note that careerists do not have a strong affective commitment; they are not striving because of a sense of belonging to the organization but because of the pursuit of something they feel is valuable beyond performance goals (Qazi et al., 2019). Thus, we propose that employees’ careerist orientation can strengthen the effects of leader narcissism on employees’ career success *via* employees’ ingratiating behavior.

## Theory framework and hypotheses

### The mediating role of employee ingratiating behavior

Ingratiation is a common impression management strategy (Bolino et al., 2008), which has been widely concerned by scholars as a political process to pursue one’s own self-interest (Ralston, 1985). Specifically, ingratiation refers to the tactics (e.g., elevating others or demeaning oneself, obeying others’ opinions, etc.) that individuals use to influence others in order to make a good impression (Jones and Pittman, 1982). Although both ingratiation and affective commitment emphasize emotional connection, the latter emphasizes more on the individual’s emotional attachment to the organization and willingness to put effort into organizational goals (Gross et al., 2021). According to dramaturgical theory, ingratiating behavior is theorized as a kind of performance behavior, which aims at satisfying the continuous occurrence of drama and gaining the favor of specific characters (Tedeschi and Melburg, 1984). In this interactive scenario, employees perform according to certain character norms (like an actor’s script). As a kind of social custom, role norms are stable social norms formed by the repeated interaction, and the role players need to obey it for performance in the interaction process. In the traditional Chinese context, employees and leaders are in an unequal position due to the authority orientation of the leadership and the emphasis on the hierarchy of identity (Farh et al., 2007). This condition provides a role norm and script for the dyadic in the theatrical interaction that leaders are in a dominant position. For employees, they are required to demonstrate the dominant position of leader through performance behavior during the interaction, and to perform in accordance with the role specification and character script. For instance, employees attribute the results of work that should be their own to leader. These acts allow employees to maintain drama throughout the interaction to avoid behavior that does not conform to the character’s requirements. Additionally, leaders with narcissistic traits need to maintain their strong self-esteem needs through flattery and praise from others (Grijalva et al., 2015), which further encourages employees to express their loyalty and obedience through ingratiation (Wu et al., 2013). As actors pursuing career success, employees will cater to narcissistic leaders who possess greater resources and power. Accordingly, we propose:

*H1*: Leader narcissism is positively related to employee ingratiation.

According to dramaturgical theory, ingratiation is an ongoing series of processes through which an interactive drama between leaders and employees takes place, requiring continuous performance by the drama participants. Employees are willing to engage in ingratiating behavior when faced with a narcissistic leader, which is consistent with role norm. In return, the leader provides the employee with career development opportunities and contributes to the career success. This is an important commitment to each other for future dramas to continue. Accordingly, the result is that leaders and employees achieve their respective benefits. Specifically, leaders win the admiration of employees, and employees receive promotion or salary increase (Goffman, 1959). Indeed, previous research also found that ingratiation can yield positive results for employees, such as promotion and salary increase (Guan et al., 2019).

There is a natural competition for individuals’ objective career growth due to scarcity of organizational resources (Mintzberg, 1985). Employees’ objective career success will inevitably lead them to obtain more exclusivity organizational resources, while others will inevitably lose a certain amount of reality and potential benefits. Employees can only achieve objective career success when they have access to the essential, scarce resources within the organization (e.g., training, Spurk et al., 2019). As mentioned above, in return, narcissistic leaders who are flattered by employees are more likely to reward employees with salary increases and coveted positions (Stern and Westphal, 2010). Li et al. (2013) pointed out that employees who ingratiate poorly are less likely to be greeted with promotions and raises than those who ingratiate well. Hence, we propose:

*H2*: Employee ingratiation mediates the relationship between leader narcissism and employee career success.

### The moderating role of employee careerist orientation

Employees with careerist orientation tend to manipulate strategies (e.g., political behavior, and deceptive behavior) to boost their careers, even if they harm the organization. They develop social relationships with their leaders not only because of their need for interpersonal relationships but also in pursuit of necessary resources (Hsiung et al., 2012). Existing studies have shown that narcissistic leaders build their audience, even treating certain employees as their “insiders” to gain admiration and be the center of attention (De Hoogh et al., 2015). Employees with careerist orientation are more likely to adopt ingratiation tactics when the narcissistic leaders are eager to be admired by employees. This exchange allows employees to realize their needs for self-promotion and career development while meeting the psychological needs of narcissistic leaders.

According to dramaturgical theory, careerist orientation is regarded as a role norm. When employees with high careerist orientation meet narcissistic leaders, they will pay more attention to the relationship with the leader (Kim et al., 2016). To leave a good impression (Adams et al., 2013), employees will demonstrate political behavior (Hsiung et al., 2012). Therefore, when employees with high careerist orientation, they are willing to choose the ingratiating behavior to respond to leader narcissism (Jones and Pittman, 1982). On the contrary, when employees have low careerist orientation, they are lack willing to manage their performance while interacting with leaders, and they do not expect that their self-image will be perfect in the leaders’ eyes, namely, reduce the level of ingratiation to leaders (Goffman, 1959). Therefore, we propose:

*H3*: Employee careerist orientation will moderate the relationship between leader narcissism and employee ingratiation. Specifically, the positive relationship between leader narcissism and employee ingratiation will be stronger when employees with higher level careerist orientation.

Further combining Hypothesis 2 and Hypothesis 3, we suggest that careerist orientation moderates the mediating effect of employee ingratiation in the relationship between leader narcissism and employees’ objective career success. Faced with the influence of leader narcissism, employees with high careerist orientation will strive to maintain their image in the leaders’ eyes by ingratiation to obtain scarce resources for achieving their career development goals (Kuyumcu and Dahling, 2014). Thus, we propose:

*H4*: Employee careerist orientation moderates the mediating effect of employee ingratiation on the leader narcissism-employee objective career success relationship, such that the mediation effect is stronger when employee careerist orientation is high.

**Figure 1:**
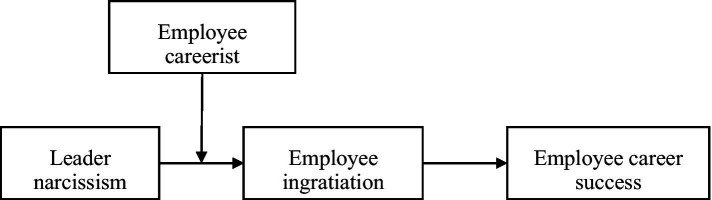
Theoretical model.

The overall theoretical model is presented in Figure 1.

## Materials and methods

### Sample and procedure

The survey sample was taken from 10 manufacturing firms in Shenzhen, Yichang, Wuhan, and Guangzhou in China. We first contacted the head of the business, and asked for his/her permission. We told the head of the business that the questionnaires were confidential and data used only for scientific research. Then, we sent questionnaires directly to participants, and the leaders were not included. A letter describing the study accompanied the questionnaire – it explained that participation was entirely voluntary and guaranteed the confidentiality of the responses. Simultaneously, to increase participation, we asked participants to complete the questionnaire during regular business hours and return it directly to our research team. All respondents signed an informed consent and agreed to participate in the study. There was no unethical behavior during the research process because this study did not involve human clinical trials or animal experiments. We collected data in two stages for this study. In the first stage (*Time 1*), the participants reported their demographic information, perceptions of their direct leader’s narcissism, ingratiation, and careerist orientation. After 1 month (*Time 2*), the same participants reported their career success. For data collection, each survey form was numbered. We sent out 400 questionnaires, recovery of valid questionnaires 299, recovery 74.8%. In the survey sample, 57.2% were female, and respondents over 25 years old accounted for 72.9%. Regarding education, 86.0% had undergraduates or above. Approximately 69.6% of respondents’ tenure was longer than 1 year.

### Measures

Part of the scale used in these studies was initially written in English. To achieve consistency in Chinese and English, we followed Brislin’s (1980) translation-back-translation procedure. Five-point Linkert scales were adopted for all relevant measures, and individuals were asked the extent to which they agreed with the statements (1 = strongly disagree, 5 = strongly agree).

Perception of leader narcissism was measured using six items (e.g., My leader is a very self-centered person) from Hochwarter and Thompson (2012) (*α* = 0.91), ingratiation using six items (e.g., I would like to express my appreciation for the way my leader handles his work) from Westphal and Bednar (2008) (*α* = 0.82), and careerist orientation using six items (e.g., The key to promotion is my interpersonal relationship, not my performance) from Chay and Aryee (1999) (*α* = 0.82). Objective career success using six items (e.g., Compared with my colleagues, my salary has been increased more rapidly) developed by Weng and Xi (2011) (*α* = 0.88). We also controlled employees’ demographic information that may affect employees’ career success (Volmer et al., 2016; Spurk et al., 2019; Van Gerven et al., 2022).

### Analytical strategy

In this study, SPSS 23.0 and Mplus 7.0 were used to analysis the sample data. Firstly, the discriminative validity among variables was tested by Confirmatory Factor Analysis (CFA), and the homologous deviation was tested by Harman’s Single-factor Test and Controlling for the effects of an unmeasured latent methods factor (ULMC) (Meade et al., 2007). Secondly, the effect value of 95% confidence interval was estimated by Bootstrapping method to test the moderated mediation effect.

## Results

### Confirmatory factor analysis and common method variance analysis

Confirmatory factor analysis was used to check the discriminant validities of the variables in our data. Meanwhile, considering that the sample size of this study was relatively small compared with the measurement items, we adopted the item parceling method in this study (Landis et al., 2000). Therefore, all of the scales, except employee career success, were constructed into three indicators, respectively, and included in the analysis. The Table 1 shows that the four-factor model yielded the best fit (*χ*2 = 71.31, *dƒ* = 38, CFI = 0.98, TLI = 0.97, RMSEA = 0.07, SRMR = 0.04), indicating the good fit of our theoretical model. Thus, we believe that the four variables involved in this paper have good distinguishing validity, confirming that they are indeed four distinct concepts.

**Table 1:** Confirmatory factor analyses.

Models	*χ^2^*	*dƒ*	*Δχ^2^*	CFI	TLI	RMSEA	SRMR
Five-factor model: SN; I; CO; CS; CMV	45.93	28	–	0.99	0.98	0.07	0.03
Four-factor model: SN; I; CO; CS	71.31	38	–	0.98	0.97	0.07	0.04
Three-factor model: SN, I + CO; CS	498.98	41	427.67^***^	0.74	0.65	0.21	0.15
Two-factor model: SN + I + CO, CS	883.11	43	811.80^***^	0.52	0.39	0.27	0.21
Single-factor model: SN + I + CO + CS	1077.12	44	1005.81^***^	0.41	0.27	0.30	0.22

*n* = 299; ***р < 0.001; SN, leader narcissism; I, ingratiation; CO, careerist orientation; CS, career success.

Based on the methods recommended by Podsakoff et al. (2003) and using Harman’s single factor test, we conducted exploratory factor analysis without rotation on all the questions in the questionnaire. The variance of the first principal component explanation was 28.48%, which was less than the 50% standard recommended by Harrison et al. (1996). In addition, controlling for the effects of an unmeasured latent methods factor is used to examine possible common method biases (Meade et al., 2007). As shown in Table 1, after adding the CMV of the five-factor model, the goodness of fit has not been significantly improved. Therefore, there is no serious problem of common method bias in this paper.

### Descriptive analysis

The means, standard deviations and correlations coefficients of the study variables are shown in Table 2.

**Table 2:** Means, standard deviations, and intercorrelations of variables.

	*M*	SD	1	2	3	4	5	6	7
1. Gender	1.57	0.50							
2. Age	1.93	0.75	−0.17^**^						
3. Education	2.91	0.63	−0.10	−0.11					
4. Tenure	2.39	1.17	−0.07	0.71^**^	−0.27^**^				
5. Leader narcissism	3.01	1.01	−0.06	0.09	−0.24^**^	0.06			
6. Employee ingratiation	3.05	0.76	−0.23^**^	0.06	0.02	0.04	0.32^**^		
7. Employee careerist orientation	2.85	0.77	−0.08	0.09	0.01	0.04	0.20^**^	0.21^**^	
8. Employee career success	2.90	0.82	−0.10	0.08	0.00	0.10	0.07	0.47^**^	0.55^**^

**р < 0.01.

### Hypothesis testing

To test the mediation hypotheses, we estimated the effect of leader narcissism on employee ingratiation (*a* path) and the effect of employee ingratiation on employee career success (*b* path), as shown in Table 3. The M2 reported that leader narcissism is positively related to employee ingratiation (*β* = 0.33, *р* < 0.001), thereby confirming Hypotheses 1. The M4 revealed that employee ingratiation is positively related to employee career success (*β* = 0.50, *р* < 0.001). These patterns are consistent with Hypotheses 2.

**Table 3:** Regression analysis models.

Variable	M1 (Employee career success)		M2 (Employee ingratiation)		M3 (Employee ingratiation)		M4 (Employee career success)	
	*β*	*t*	*β*	*t*	*β*	*t*	*β*	*t*
Gender	−0.17	−1.4	−0.42	−3.77^***^	−0.29	−3.49^***^	0.04	0.37
Age	−0.03	−0.28	−0.05	−0.50	−0.05	−0.62	−0.01	−0.05
Education	0.07	0.68	0.14	1.50	0.08	1.16	0.01	−0.01
Tenure	0.10	1.40	0.05	0.72	0.03	0.62	0.08	1.20
Leader narcissism (LN)	0.07	1.23	0.33	5.89^***^	0.25	5.73	−0.09	−1.58
Employee ingratiation							0.50	8.87^***^
Employee careerist orientation (CO)					0.11	2.13^*^		
LN × CO					0.12	2.42^*^		
*R* ^2^	0.02		0.16		0.19		0.23	
*F*	1.43^***^		10.77^***^		9.65^***^		14.62^***^	

*р < 0.05, ***р < 0.001.

To test the mediation effect of employee ingratiation, we estimated the indirect, direct, and total effects along with their 95% confidence intervals (CI) using the bootstrapping procedure based on PROCESS macro (Hayes, 2017). As shown in Table 4, the significant influence of leader narcissism on employee career success can be attributed to its indirect impact *via* employee ingratiation rather than its direct impact. Specifically, the indirect effects of leader narcissism on employee career success *via* employee ingratiation was significant (estimate = 0.16, *р* < 0.001), and with the bootstrap set to 5,000 times, 95% confidence interval (CI) [CI = 0.09, 0.24] did not include zero. Thus, these results support Hypothesis 2.

**Table 4:** Mediation effect analysis.

	Effect	Boot SE	95% Boot LLCI	95% Boot ULCI
Total effect	0.07	0.06	−0.04	0.19
Direct effect	−0.09	0.05	−0.20	0.02
Indirect effect	0.16	0.04	0.09	0.24

Bootstrap = 5000.

**Figure 2:**
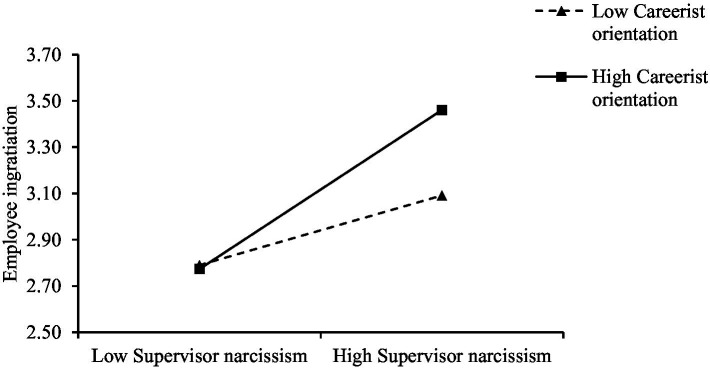
Moderating effect of employee careerist orientation on the relationship between leader narcissism and employee ingratiation.

The regression results of M3 in Table 3 indicate that the interaction term of leader narcissism and employee careerist orientation significantly predicted employee ingratiation (*b* = 0.12, *р* < 0.05). To explain the interactive effect clearly, we constructed an interaction plot following the procedures recommended by Aiken and West (1991). The results are shown in Figure 2, which reveals that the conditional effect of leader narcissism on employee ingratiation at the moderator (employee careerist orientation) level was low and high (±1 SD from the mean). Simple slope test results show that the effect of leader narcissism on employee ingratiation was more pronounced and positive with high (*b* = 0.34, *р* < 0.001, 95% CI = [0.23, 0.47]) rather than low (*b* = 0.15, *р* < 0.01, 95% CI = [0.05, 0.25]) employee careerist orientation. Thus, Hypothesis 3 was supported.

We followed Hayes (2017) recommended method of testing for moderated mediation and used the PROCESS macro to estimate the conditional indirect effects of leader narcissism on employee career success with varying levels of employee careerist orientation. As shown in Table 5, the indirect effect of leader narcissism on employee career success was weak at low levels of employee careerist orientation (−1 SD estimate = 0.08; 95% CI = [0.02, 0.15]), but was substantially stronger at high levels of employee careerist orientation (+1 SD estimate = 0.18; 95% CI = [0.11, 0.26]). The results further confirm that employee careerist orientation will affect the degree of employees’ ingratiation with leaders, rather than giving up ingratiation. Moreover, the index of moderated mediation reveals the indirect effects at various conditional values of the moderator (Index estimate = 0.07; 95% CI = [0.01, 0.13]). As shown in Table 5, all indexes for the moderated mediation effects are statistically significant and not include zero. Thus, Hypothesis 4 was supported.

**Table 5:** Indirect effects of leader narcissism-employee ingratiation-employee career success.

	Effect	Boot SE	Boot LLCI	Boot ULCI
CO low-1 SD	0.08	0.03	0.02	0.15
CO mean	0.13	0.03	0.08	0.19
CO High +1 SD	0.18	0.04	0.11	0.26
CO index	0.07	0.03	0.01	0.13

## Discussion

By constructing a moderated mediation model to explain how and when leader narcissism promotes employees’ objective career success, we extend the application of dramaturgical theory and contribute to the literatures of leader narcissism and employee career development. Specifically, we demonstrate that leader narcissism motivates employees’ ingratiation, which facilitates employees’ career success, especially when employees with higher level careerist orientation.

### Theoretical implication

There are several outstanding theoretical contributions that deserve attention. First, we expand the application of dramaturgical theory by using it to explain the promotion of leader narcissism on employees’ objective career success. Social exchange theory or signaling theory (Golden and Eddleston, 2020) is the main theoretical framework to explain how employees interact with their leaders in pursuit of career success in previous literatures. Most of these studies focused on the perspective of leaders and asserted that employees interact with leaders according to specific situations. In line with this logic, if we want to reduce the negative influence of leaders, we can only reduce the interaction frequency between leaders and employees which posted a difficult task. However, our study emphasized the interaction process between employees and their leaders and the employees’ experience based on dramaturgical theory, which provided a new perspective and theoretical explanation for the study of employee career success.

Second, we uncovered the mechanism of how leader narcissism promotes employees’ career success by introducing employee ingratiation as a mediating variable. While discussing the effect of leader narcissism, most extant literatures focused on the direct adverse effects of leader narcissism in the workplace, such as organizational innovation (Cragun et al., 2020). To explore the potential value of leader narcissism, this study introduced employees’ ingratiation as a mediating variable to uncover the internal mechanism of leader narcissism and employee objective career success. Our findings not only support the positive effects of leader narcissism, but also provides more generalizable conclusions related to internal impact mechanism of leader narcissism and objective employee career success. Thus, our study provides a novel way to explain existing employee objective career success as well as insight into how leader narcissism relates to previous theory.

Third, this study enriches the research on career development by examining the moderating effect of careerist orientation. Our results showed that careerist orientation not only moderates the relationship between leader narcissism and employee ingratiation but also moderates the indirect effects of leader narcissism on employee career success through employee ingratiation. This reveals that actor’s career goals should be considered to explain the influence of leader narcissism on employees’ objective career success with dramaturgical theory. Namely, considering varying levels of careerist orientation, the impact of leader narcissism on employees’ objective career success varies. Additionally, previous studies have generally described careerist orientation negatively affects work attitudes on account of it sensitizes individuals to the current negative aspects of their jobs (Chay and Aryee, 1999), our study provides a new perspective for the research field of careerist orientation.

### Practical implication

First, our study can be helpful for organizations looking to understand more about the favorable of leader narcissism. The adverse effects of leader narcissism have been confirmed by many scholars (Grijalva et al., 2015). However, young people must adapt to these leaders to pursue objective career success. From the perspective of personal factors, employees with faster career growth have higher career satisfaction, clearer career self-concept and higher career efficacy and commitment. From the perspective of the impact of individuals on the organization, the faster the career growth, the higher the organizational commitment, the more organizational citizenship behavior and lower turnover tendency and behavior. At the same time, organizations should also pay attention to the narcissistic leader to allocate organizational resources fairness and rationality.

Second, while ingratiation can be a forceful tool for personal objective career success, employees should be aware that leadership style or preferences is the determining factor of ingratiation, and failed ingratiation can be detrimental to employees’ career success. Employees who use ingratiation to achieve career success and should know that such efforts are likely not appreciated by your leader. Hence, employees should also attempt to substantially develop their real capabilities to enhance career performance, thereby achieve career success.

Third, employees who are ambitious should be monitored. This study found that employees with careerist orientation often exploit the potential effects of leadership narcissism. As a result, their ingratiation is directly related to objective career success. On the one hand, leaders should strengthen their understanding of themselves to avoid narcissism and prevent being exploited by employees with careerist orientation. On the other hand, encouraging these employees to focus on job performance rather than relationships to preventing the potential effects of leader narcissism.

### Limitation and future direction

Despite the strengths, the present study is not without limitations. First, because the current research focuses on employees’ responses toward leader narcissism, we collected self-reported data from employees, and especially we used employee perceptions to measure leader narcissism, which may give rise to the risk of common method bias (Podsakoff et al., 2003). To minimize CMV, we separated the measures between leader narcissism and employee career success. Furthermore, we controlled for the employees’ demographic information measured at Time 1. In the future, data collection can be carried out in combination with experimental methods and multi-source evaluation methods. Second, based on dramaturgical theory, we explored the impact of leader narcissism on employees’ objective career success through employee ingratiating behavior and under the condition of the employee’s careerist orientation. Future research can excavate other variables (e.g., leadership style, and career adaptability) that affect employees’ career success and further deepen the theoretical understanding of employees’ career development. For example, if the leader is pseudo-transformational, it means that the leader and organizational goals are incompatible, which is likely to hinder employees’ career development or even cause counterproductive work behavior. And future research may be also benefit from control other different impression management strategies (e.g., self-promotion and supplication) to prove the unique effect of ingratiation. Finally, as a means of impression management, this study examined the mechanism role of employee ingratiation with dramaturgical theory, and future research should examine the influence of employees’ different impression management strategies on employee and leaders.

## Data availability statement

The raw data supporting the conclusions of this article will be made available by the authors, without undue reservation.

## Author contributions

ZD, ML, and LQ designed and adopted the study, and wrote the manuscript. HW, PZ, and WL helped to perform the analysis with constructive discussions. All authors contributed to the manuscript revision, read, and approved the submitted version.

## Funding

This project was supported by the National Natural Science Foundation of China (Grant Nos. 72072066 and 72172157) and Program for the Humanity and Social Science Youth Foundation of the Ministry of Education of China (Grant No. 19YJC630165).

## Conflict of interest

The authors declare that the research was conducted in the absence of any commercial or financial relationships that could be construed as a potential conflict of interest.

## Publisher’s note

All claims expressed in this article are solely those of the authors and do not necessarily represent those of their affiliated organizations, or those of the publisher, the editors and the reviewers. Any product that may be evaluated in this article, or claim that may be made by its manufacturer, is not guaranteed or endorsed by the publisher.
